# Multifunctional REE Selective Hybrid Membranes Based on Ion-Imprinted Polymers and Modified Multiwalled Carbon Nanotubes: A Physicochemical Characterization

**DOI:** 10.3390/ijms26157136

**Published:** 2025-07-24

**Authors:** Aleksandra Rybak, Aurelia Rybak, Sławomir Boncel, Anna Kolanowska, Waldemar Kaszuwara, Mariusz Nyc, Rafał Molak, Jakub Jaroszewicz, Spas D. Kolev

**Affiliations:** 1 Department of Physical Chemistry and Technology of Polymers, Faculty of Chemistry, Silesian University of Technology, Strzody 7, 44-100 Gliwice, Poland; 2 Department of Electrical Engineering and Industrial Automation, Faculty of Mining, Safety Engineering and Industrial Automation, Silesian University of Technology, 44-100 Gliwice, Poland; aurelia.rybak@polsl.pl; 3 Department of Organic Chemistry, Bioorganic Chemistry and Biotechnology, Faculty of Chemistry, Silesian University of Technology, 44-100 Gliwice, Poland; slawomir.boncel@polsl.pl; 4 Institute of Chemistry, Faculty of Science and Technology, University of Silesia, 40-007 Katowice, Poland; anna.kolanowska@us.edu.pl; 5 Faculty of Materials Science and Engineering, Warsaw University of Technology, 02-507 Warszawa, Poland; waldemar.kaszuwara@pw.edu.pl (W.K.); mariusz.nyc@pw.edu.pl (M.N.); rafal.molak@pw.edu.pl (R.M.); jakub.jaroszewicz@pw.edu.pl (J.J.); 6 School of Chemistry, The University of Melbourne, Melbourne, VIC 3010, Australia; s.kolev@unimelb.edu.au; 7 Department of Chemical Engineering, The University of Melbourne, Melbourne, VIC 3010, Australia; 8 Faculty of Chemistry and Pharmacy, Sofia University “St. Kl. Ohridski”, 1 James Bourchier Blvd., 1164 Sofia, Bulgaria

**Keywords:** ion-imprinted biopolymers, functionalized MWCNTs, multifunctional hybrid membranes, magnetic properties, thermal stability, mechanical properties

## Abstract

A novel type of multifunctional hybrid membranes combining modified chitosan, functionalized multi-walled carbon nanotubes (MWCNTs), and rare earth element ion-imprinted polymers (REEIIPs) were designed and characterized. The synthesized materials were characterized by thermogravimetric analysis (TGA), scanning electron microscopy (SEM), vibrating sample magnetometry (VSM), X-ray diffraction (XRD), X-ray micro-tomography, and Fourier transform infrared spectroscopy (FTIR). The hybrid membranes were also studied in terms of their mechanical and rheological properties. The key element of the proper preparation of hybrid membranes using the casting method in an external magnetic field was to synthesize membrane components with appropriate magnetic properties. It was found that they showed tunable weak ferromagnetic properties, and the increase in modified nanotube addition caused the rise in the membrane’s saturation magnetization, which for Nd-selective hybrid membranes reached 0.44 emu/g. Also, the increase in thermooxidative stability was noted after introducing functionalized nanotubes into polymer matrices, which, in the case of Gd-selective membranes, were stable even up to 730 °C. The rise in the modified MWCNT addition and selection of appropriate REE ion-imprinted polymers improved mechanical (Rm and E values increase even twice) and rheological parameters (almost double growth of E′ and E″ values) of the tested membranes. Synthesized hybrid membranes showed a high rejection of matrix components and an increase in retention ratio with rising MWCNT-REEIIP addition, ultimately reaching 94.35%, 92.12%, and 90.11% for Nd, Pr, and Gd, respectively. The performed analysis confirmed homogeneous dispersion, phase compatibility, network integration, formation of a complex 3D microstructure, and improved operational stability of created hybrid membranes, which is significant for their future applications in Nd, Pr, and Gd recovery from coal fly ash extracts.

## 1. Introduction

Nowadays, the development of modern technologies requires access to stable and reliable sources of raw materials. This is also the situation related to the production of electronic devices and the development of clean energy technologies, and the defense, optics, automotive, agriculture, medicine, and energy industry, among others [1], where one of the most important raw materials are rare earth elements (REEs). Especially due to their unique chemical, catalytic, physical, magnetic, and luminescent properties, they are used in an increasing range of modern products and technologies [1,2]. They are obtained from the ores of these metals, the main deposits of which are in China, which has close to a monopoly on their production [1]. In addition, their production is carried out using conventional mining techniques, which, in turn, is associated with significant energy consumption and the generation of huge amounts of toxic waste and acidic sewage. Therefore, it is extremely important to search for new sources of these elements, especially considering the requirements of the closed-loop economy and the need to reduce the amount of industrial waste and use them as secondary sources. In the case of REEs, coal fly ash can be an excellent source due to their presence in the ash composition, the availability of this waste product, and the need to reduce numerous problems related to its storage [2,3].

Many chemical, physical, and biological methods are used for the recovery of REEs from coal fly ash [4,5], the most promising of which are chemical methods based on the extraction of REEs using various extracting solutions, such as HCl, HNO_3_, H_2_SO_4_, or NaOH [2,3,4,5,6,7]. Using these methods, extracts of complex composition, extreme pH, and high concentrations of other components of the coal fly ash matrix (Al, Si, Fe) extracted together with the separated REEs are obtained [8]. In addition, the recovery of a mixture of many REEs can also be problematic due to their chemical similarity and potential problems with their separation. Therefore, it is important to introduce highly selective methods, such as membrane and adsorption techniques [9], based on advanced materials that can work in extreme environments [10,11,12,13]. For the separation of REEs from extracts, various membrane techniques were used, such as reverse osmosis (RO), nanofiltration (NF), emulsification liquid membrane (ELM), and hollow fiber liquid membranes (HFLM) based on various types of membranes [14,15,16]. REE enrichment above 90% was obtained for all membrane-based techniques. However, there are still many issues that need to be resolved, such as limited efficiency, fouling, and the short life of membranes. Despite these issues, the membrane techniques are a good alternative to conventional methods (ion exchange and chemical precipitation) for separating REEs from extracts and have the potential for further development in the future, especially based on advanced materials with high selectivity and affinity for REE ions.

In recent years, polymeric materials operating on the principle of a lock and key mechanism, imitating the action of natural receptors such as antibodies or enzymes in biological systems, have become highly promising in the recovery of trace amounts of various compounds [17]. For this purpose, molecular imprinted polymers (MIPs) or ion-imprinted polymers (IIPs) are used, containing cavities in the polymer matrix matching the structure of the target substances. These materials are highly selective and can be used to concentrate or recover target compounds from samples with complex matrices. They can be obtained by the imprinting technique, during which a template molecule or ion is dissolved in a suitable solvent, the so-called porogen, together with functional monomers (capable of polymerization), between which a pre-polymerization complex is formed. Then, after adding a cross-linking agent and carrying out the polymerization reaction, a cross-linked polymer is formed, from which, in the next step, the template ion or molecule is removed, and three-dimensional cavities are created with a shape corresponding to the template and capable of repeatedly adsorbing and desorbing it with high selectivity [18]. In recent years, such materials have been most often used in applications such as beds in the SPE technique to concentrate Eu, Ce, Nd, Dy, and Y from wastewaters, human serum, and plasma, and as sensors [17,19], to improve the specific identification of artemisinin [20], for Cu^2+^ detection [21], and for the capture and purification of hemoglobin [22], among others.

In the literature, there are various classifications of IIP synthesis methods, the most popular of which divides them into four groups, namely (1) cross-linking of linear polymer chains carrying groups capable of forming complexes with ions, (2) chemical immobilization of ligands with a vinyl moiety in the presence of a cross-linking agent, (3) surface imprinting involving a ligand with or without a vinyl group, and (4) entrapment of a non-vinylated ligand inside the polymer network [11]. In the case of IIPs for REEs, the fourth method proved to be the most effective [23].

Several components are needed to obtain IIPs by the fourth method, such as a template ion, which is usually a metal ion [24], functional monomers containing vinyl groups, such as 1-vinylimidazole (1-VI), 2-hydroxyethyl methacrylate (HEMA), methyl methacrylate (MMA), acrylamide (AAm), methacrylic acid (MAA), 4-vinylpyridine (4-VP), and acrylic acid (AA) [24,25,26,27,28,29,30,31,32]. In addition, the physical entrapment of various non-vinylated ligands (such as dithizone (DZ), diphenylcarbazide (DPC), and 8-hydroxyquinoline (8-HQ)) in the IIP networks generated by copolymerization of vinylated ligands with a cross-linking agent [31,32] is also used. Other key IIP components are cross-linkers used to stabilize the IIP network and provide appropriate mechanical stability, flexibility, mass transfer efficiency, appropriate arrangement, and a number of recognition sites per unit mass of the IIP. The cross-linkers can be classified into two groups, i.e., those containing vinyl groups that can react with functional monomers (e.g., ethylene glycol dimethacrylate (EGDMA), poly(ethylene glycol) diacrylate (PEGDA), *N*,*N*′-methylenebisacrylamide (MBAAm), trimethylolpropane trimethacrylate (TMPTMA), and divinylbenzene (DVB)) and those that are used for cross-linking linear or hyperbranched polymers (e.g., glutaraldehyde (GA) and epichlorohydrin (ECH)) [24,31,32]. The synthesis of IIPs also involves the use of initiators, the most commonly used among them being azobisisobutyronitrile (AIBN), and porogens, which provide a suitable polymerization reaction environment with an appropriately selected polarity and dielectric constant (e.g., methanol, toluene, dimethyl sulfoxide (DMSO), *N*,*N*-dimethylformamide (DMF), acetonitrile, acetic acid (CH_3_COOH), trichloromethane (CHCl_3_), etc.). In the final step of the IIP development, it is necessary to remove template ions or molecules using suitable reagents, such as HCl, H_2_SO_4_, HNO_3_, or ethylenediaminetetraacetic acid (EDTA) and thiourea in the case of using functional monomers that are unstable in an acidic environment [32].

In addition to the abovementioned methods, used most frequently for the synthesis of IIPs, there are some others involving the use of sol–gel, surface imprinting, stimuli-responsive imprinting, dual template imprinting, a combination of various ion imprinting technologies, and free radical polymerization [24]. Free radical polymerization is used more often than the other methods mentioned above, and it may involve bulk, precipitation, suspension, or emulsion polymerization [24,32]. An extremely important issue in the case of IIPs is to obtain selective materials with adequate chemical, thermal, and mechanical strength, which can be used under different conditions, and this requires the introduction of appropriate modifications.

Another promising method of REE recovery is magnetic solid-phase extraction (MSPE), during which the target species are adsorbed on magnetic adsorbents (e.g., Fe_3_O_4_ [33], Fe_3_O_4_@SiO_2_@polyaniline–graphene oxide composite [34]), which can be easily separated from the solution by applying a magnetic field. In turn, adsorbents based on carbon nanotubes have also become highly promising in the recovery of trace amounts of metal ions, which are most often used for the analysis of Pb, Au, Rh, Mn, Fe, Cu, and REEs in samples such as mineral water, serum, synthetic seawater, garlic, and rocks [34,35,36]. However, due to dispersion problems in solvents or polymer matrices, these materials require further modifications by covalent or non-covalent functionalization, such as poly(acrylic acid) (PAA) introduction [19].

The aim of the study described in this paper and key novelty was to synthesize novel, previously unprepared, and unused Nd, Pr, and Gd ion-imprinted composite polymers and modified magnetic multi-walled carbon nanotubes (MWCNTs) functionalized with these REE ion-imprinted polymers (REEIIPs), and to prepare hybrid membranes based on these new materials. The obtained polymers, MWCNT fillers, and membranes were characterized in terms of their chemical, magnetic, mechanical, rheological, thermooxidative, and structural properties on one hand and their significance for the recovery of Nd, Pr, and Gd ions from synthetic extracts on the other. As part of the synthesis of the newly developed REE selective hybrid membranes, the method of membrane casting in an outer magnetic field, proposed by the authors, was used [37,38,39,40,41], which ensured adequate dispersion of the inorganic filler particles in the polymer matrix.

## 2. Results and Discussion

### Characteristics of Synthesized REEIIPs and Hybrid Materials

**Figure 1:**
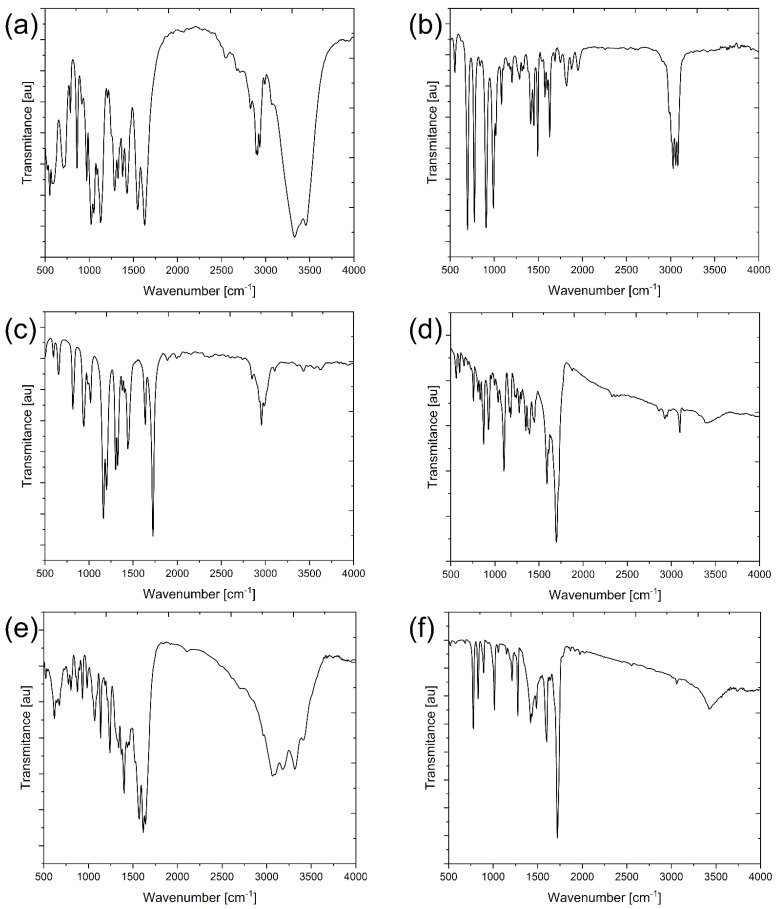

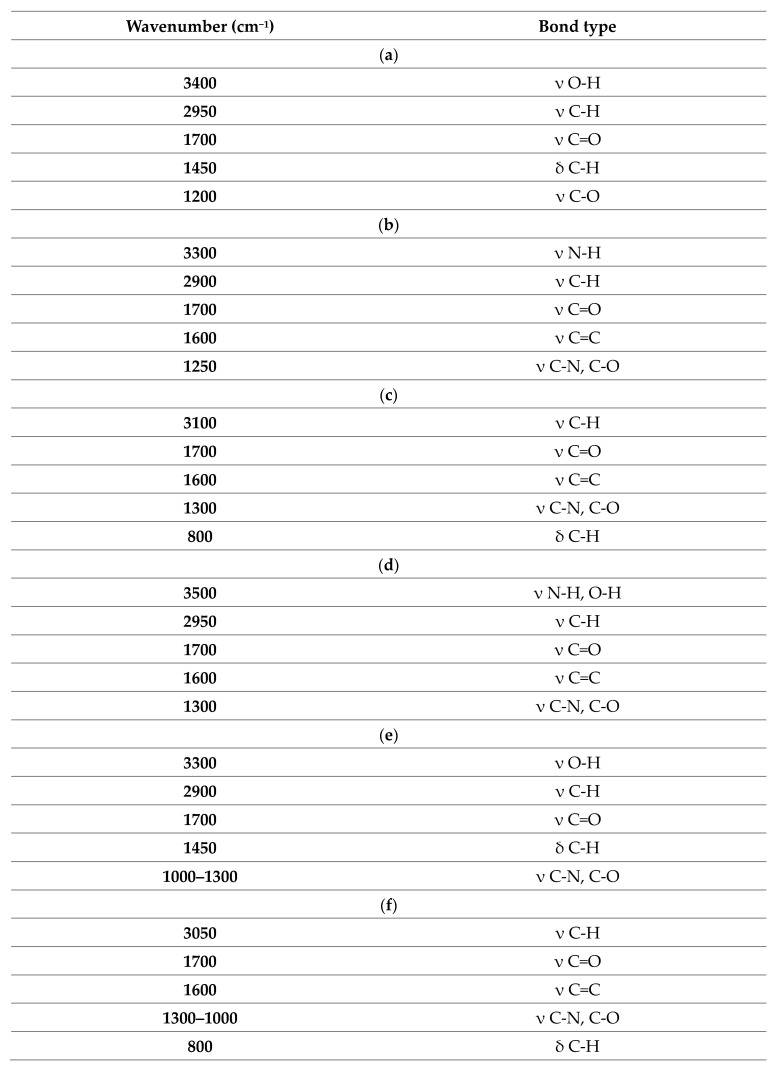
FTIR spectra of the following: (**a**) modified chitosan, (**b**) NdIIPs, (**c**) PrIIPs, (**d**) GdIIPs, (**e**) MWCNT—PrIIPs, and (**f**) MWCNT—GdIIPs.

Synthesized polymers and nanotubes were analyzed with several techniques. Among others, the FTIR was carried out, and the corresponding spectra are presented in Figure 1. Many characteristic absorption peaks confirming the chemical structure of the synthesized polymers could be observed. For example, in the modified chitosan, the interaction between the chitosan matrix and the aldehyde through the formation of new imine linkages was confirmed by the band at 1621 cm^−1^. A shift in the position of characteristic bands when compared to the pure chitosan that could confirm the formation of chitosan Schiff base could also be observed, i.e., the characteristic bands at 3361 and 3291 cm^−1^ corresponding to N-H and O-H stretching were shifted in the Schiff base-like product to 3462 and 3331 cm^−1^. The absorption bands at 2921 and 2877 cm^−1^, attributed to the C-H symmetric and asymmetric stretching, were shifted to 2945 and 2890 cm^−1^. These bands are generally characteristic for polysaccharides. The residual *N*-acetyl groups were confirmed by the band at 1621 cm^−1^ (C=O stretching at amide I) and a band shift from 1329 to 1283 cm^−1^ (C-N stretching of amide II). The band shift from 1589 to 1621 cm^−1^ corresponds to the N-H bending in the product. The shift in the absorption band from 1153 to 1136 cm^−1^ can be attributed to the stretching of the C-O-C bridge, while the bands at 1283 and 1186 cm^−1^ could correspond to C-N stretching after the formation of the Schiff base. The absorption at 1431 cm^−1^ is associated most probably with the hydroxy groups of chitosan. The band at 1621 cm^−1^ could be assigned to C=C bonds associated with the skeletal vibrations. In our case, the absorption bands between 1500 and 1750 cm^−1^ correspond to the C=N bonding, so the band at 1621 cm^−1^ could be associated with this bonding.

It could be seen that the FTIR spectra of the REEIIPs are similar because these materials were synthesized with the same precursors and by the same methodology. However, some differences could be observed, especially connected with various ternary complexes. FTIR spectra confirmed the successful incorporation of functional groups characteristic of modified chitosan and REEIIPs (Nd, Pr, Gd).

For all of the REEIIP samples, it was found that the REE ions were bound by nitrogen atoms, due to the change in the C=N band around 1690 cm^−1^ for DCQ [42], to 1633 cm^−1^ for NdIIPs, to 1643 cm^−1^ for PrIIPs, and to 1595 cm^−1^ for GdIIPs. Also, the peak around 1700 cm^−1^ could confirm the presence of a hydrogen bond bridge and the ternary complex. However, there are some changes in the position of characteristic bands that could be related to the creation of a cross-linked structure and preorganization of the ligands by the template. The absence of bands in the region of 1683 cm^−1^ indicated the absence of a vinyl group, confirming the successful polymerization.

It was also possible to detect in each polymer the presence of polystyrene absorption bands associated with the stretching of the C=O group at 1633 cm^−1^ for NdIIPs, at 1643 cm^−1^ for PrIIPs, and at 1595 cm^−1^ for GdIIPs, and the C-O group at 1114 cm^−1^ for NdIIPs, at 1464 cm^−1^ for PrIIPs, and at 1100 cm^−1^ for GdIIPs. The transformation into the REEIIPs was confirmed by the presence of C-N aromatic amine stretching bands at 1214, 1305, and 1276 cm^−1^ for Nd, Pr, and GdIIPs, respectively.

Figure 1e,f show exemplary FTIR spectra for MWCNTs modified with PrIIPs and GdIIPs, respectively. The spectrum in Figure 1e shows multiple broad and overlapping absorption bands indicative of a chemically diverse surface. The most prominent feature is a broad absorption band centered around 3300–3400 cm^−1^, commonly associated with O–H stretching vibrations, which may originate from hydroxyl groups introduced during the oxidative pretreatment or polymerization steps. This band may also include N–H stretching from amide or amine groups, indicating the incorporation of nitrogenous functionalities, possibly through monomers used during polymer imprinting. The region between 2800 and 3000 cm^−1^ reveals a weaker, yet discernible, set of bands corresponding to aliphatic C–H stretching vibrations, suggesting the presence of saturated alkyl chains from the polymer backbone. These features are characteristic of methylene (-CH_2_-) and methyl (-CH_3_) groups, likely derived from the vinyl group in monomers. The sharp band near 1650 cm^−1^ is typically attributed to C=O stretching in amide or carboxylic acid groups. Bands around 1570–1620 cm^−1^ could correspond to aromatic C=C stretching or the asymmetric stretching of carboxylate groups. Absorptions in the 1000–1300 cm^−1^ range are assigned to C–O, C–N, or C–O–C stretching, indicative of ether linkages. The spectral complexity in this fingerprint region confirms that the pristine CNTs were successfully functionalized with a chemically rich polymer matrix capable of molecular imprinting. The broad bands and overlap suggest a heterogeneous environment typical of non-crystalline polymer networks. Figure 1f of MWCNT-GdIIPs displays a more defined and sharper IR spectrum, indicating a higher degree of chemical order or more consistent functional group incorporation. Most notably, the spectrum exhibits a strong and narrow absorption band around 1720 cm^−1^, which is characteristic of C=O stretching in ester or carboxylic acid groups. This is a key signature for the successful grafting of functional monomers to the CNT surface. Compared to the pristine-imprinted sample, the O–H/N–H stretching region around 3400 cm^−1^ appears narrower and less intense, possibly indicating fewer free hydroxyl groups on CNTs or more extensive hydrogen bonding within the polymer network. The C–H stretching region (2800–3000 cm^−1^) remains present, confirming the aliphatic C–H stretching vibrations, suggesting the presence of saturated alkyl chains from the polymer backbone. Multiple peaks are evident near 1200–1450 cm^−1^, which can be attributed to C–N, C–O, and C–C stretching as well as CH_2_/CH_3_ bending vibrations. The region around 1600 cm^−1^ also likely includes N–H bending or aromatic C=C stretching, reflecting the imprinted polymer’s chemical framework. The increased spectral resolution and the pronounced carbonyl peak confirm that the grafting approach has led to a well-defined and chemically robust imprinted surface on the CNTs. These structural features enhance the material’s potential for selective ion recognition, likely improving the sensitivity and selectivity of obtained materials.

The synthesized membranes were also studied by various techniques, like XRD, TGA, SEM, X-ray micro-tomography, VSM, DMA, and the mechanical technique.

**Figure 2:**
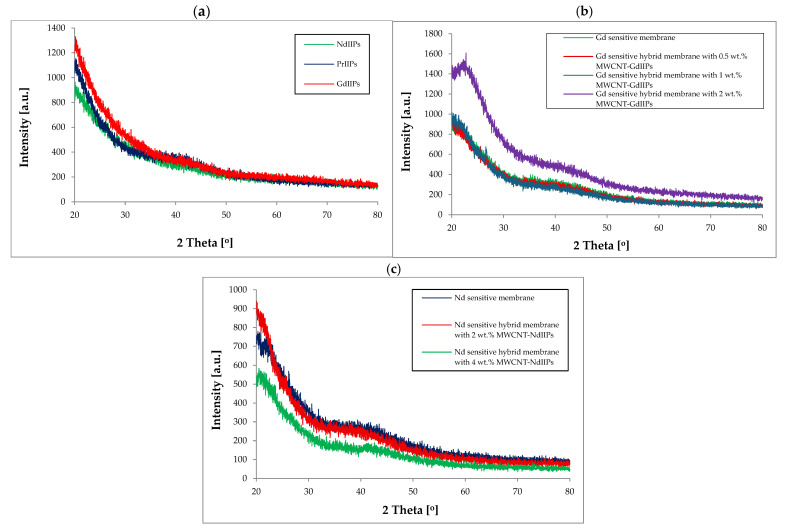
XRD spectra for Nd, Pr, and Gd sensitive adsorption membranes: (**a**) REEIIP dense membranes, (**b**) hybrid membranes with 0.5 wt.%, 1 wt.%, and 2 wt.% addition of MWCNT-GdIIPs, and (**c**) hybrid membranes with 2 wt.% and 4 wt.% addition of MWCNT-NdIIPs.

Selected results of the XRD analysis of pure and hybrid membranes with additions of various MWCNTs are presented in Figure 2.

**Figure 3:**
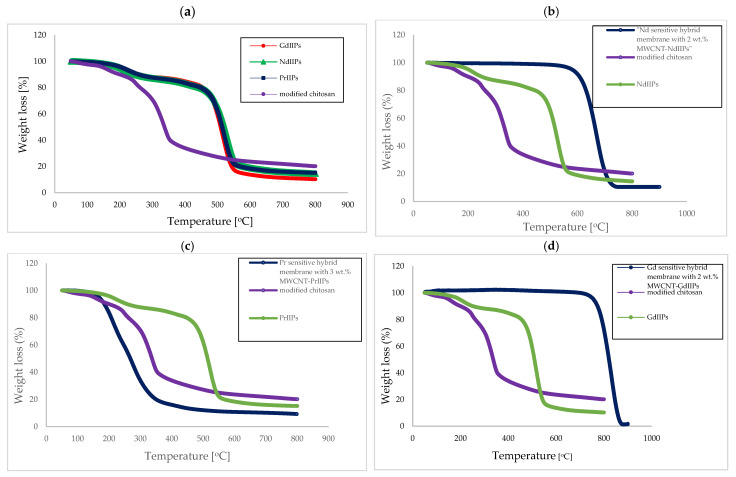
(**a**–**d**) TGA curves of polymer membranes made of modified chitosan, REEIIPs, and hybrid membranes based on synthesized polymers with various modified MWCNT-REEIIP additions.

The synthesized polymers were also characterized by X-ray diffraction (XRD), and the results are presented in Figure 2a. Here, we can see that this is a typical structure of semicrystalline polymers with some weak peaks associated with the introduced REE ions, usually around the angle 2 theta 42–45°, that indicates reduction in crystallinity due to polymer chain crosslinking and additive dispersion. In the case of REE sensitive adsorption membranes (Figure 2b,c), characteristic broad peaks originating from the polymer and graphite were found. The shape of these peaks changes slightly, e.g., after introducing larger amounts of additives, especially in the case of MWCNTs modified with REEIIPs, they become sharper, and an additional small overlapping peak appears. The decrease in these peaks’ intensity could be caused by structural disorder and improved matrix homogenization. In the case of membranes modified by the GdIIPs and the introduction of a larger addition of modified MWCNTs, the movement of peak maximum with the greatest intensity in the direction of a higher angle of 2 Theta was observed. In addition, a slight increase in the intensity of this peak was also observed as the addition of modified nanotubes increased for all types of tested adsorption membranes. The TGA was conducted for all synthesized membranes (Figure 3).

The TGA curve for modified chitosan (Figure 3a) contains a few stages. There is a small weight decrease (about 2%) in the range between 50 and 93 °C, which could be related to the moisture release. In the next step, i.e., between 245 °C and 370 °C, a weight loss of 47% was observed, which could be caused by the loss of heterocyclic rings and phenyl groups attached to the backbone of chitosan during depolymerization [43]. In the last stage, the recorded weight loss was about 16%, leaving, eventually, a residue amounting to approximately 20 wt.%. This behavior indicated that up to 800 °C, not the whole modified chitosan was decomposed, and the observed weight loss was caused by the decomposition of the unreacted amino groups. Comparing the abovementioned results with the literature results for the pure chitosan (i.e., a weight loss of 11.5% between 27 and 105 °C, 51% between 255 and 363 °C, 22% in the third stage, and the residual mass of 8%), it can be concluded that after modification, the thermal stability of the chitosan had improved.

The TGA results for the REEIIP (Figure 3a) indicate several stages. The first stage (49–124 °C) can be assigned to the evaporation of the entrapped solvent. The second stage (124–270 °C) corresponds to the pyrolysis of quinolinol (the melting point of 5,7-dichloroquinoline-8-ol is 180 °C). And the next stage (270–570 °C) relates to the further decomposition of the REE (5,7-dichloroquinoline-8-ol)_3_ complex and polymer material. The residual mass was in the 10–20% range.

In the case of Pr selective membranes with the addition of MWCNT modified with PrIIPs, a faster decomposition of the polymer matrix was observed. However, they were still stable until 250 °C, considered to be adequate for their potential application as adsorption membranes for REE recovery. However, in the case of other membranes selective for Nd and Gd, the tendency was opposite. Namely, their thermooxidative stability increased, and the temperatures at which decomposition was observed shifted to higher values. The Nd-selective membranes were characterized by enhanced thermal properties compared to the modified chitosan, even for pure NdIIPs, which were stable until 480 °C. After the introduction of modified MWCNTs (Figure 3b–d), namely with ion-imprinted polymer NdIIPs, their thermooxidative stability improved, and they were stable up to 590 °C. An increase in the thermooxidative stability after the introduction of modified MWCNTs was also observed for the Gd-sensitive membranes, i.e., the membranes with the addition of 2 wt.% MWCNT-GdIIPs were stable up to 730 °C. So, these hybrid membranes were characterized by enhanced thermal stability, which increased with the rise in MWCNT addition.

**Figure 4:**
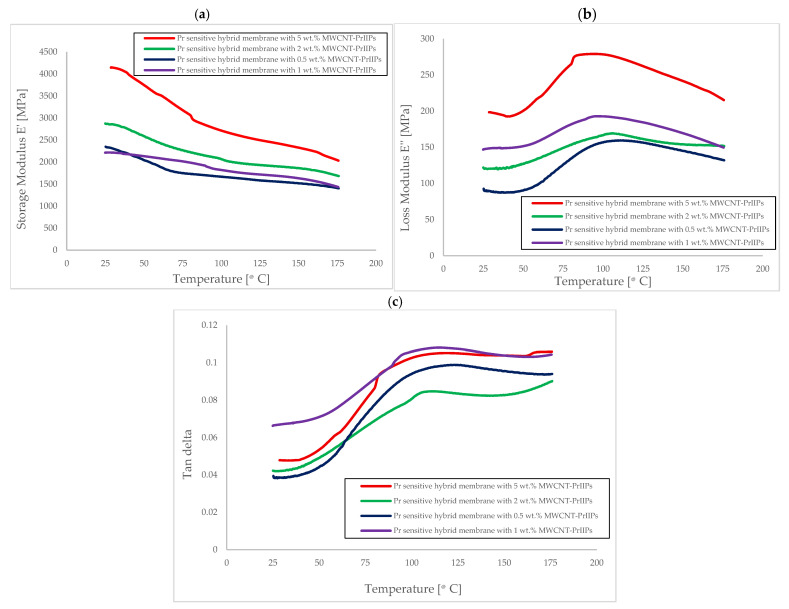
Variation in (**a**) storage modulus E′, (**b**) loss modulus E″, and (**c**) loss factor tan δ as a function of the temperature for Pr sensitive membranes with various modified MWCNT-PrIIP additions.

The newly developed membranes were studied by rheological analysis using the dynamic mechanical method (DMA), which allowed us to characterize their viscoelastic properties in wide temperature ranges. Three parameters were used to describe the rheological properties, namely the storage modulus, E′, the loss modulus, E″, and the loss coefficient tan δ (Figure 4). The temperature dependencies of E′, E″, and tan δ provided information on the mobility of polymer chains. At lower temperatures, local movements related to low-amplitude motions of chain segments or within the lateral groups (β relaxations) could be observed. At higher temperatures, however, molecular movements related to glass transition (α relaxations) were observed, and their occurrence could be associated with substantial changes in the mechanical properties of materials. As can be seen in Figure 4b,c, the relaxation process is associated with peaks near 95–110 °C for all hybrid membranes. However, the tan δ peak was observed at higher temperatures in the range from 110 to 120 °C. Figure 4a shows significantly higher values of E′ for membranes with a larger MWCNT content. This phenomenon could be caused by the higher number of nanoparticles in the polymer matrix, whose stiffness was much higher than that of the polymer matrix. With the increase in nanoparticle addition, the distance between them decreased, thus leading to the formation of more complex 3D microstructures with higher storage modules E′ (almost twice). The modification of chitosan with aldehyde and the introduction of second REEIIPs resulted in a decrease in the glass transition temperature (from 140 °C for chitosan [44] to around 110 °C). Additionally, the introduction of modified MWCNTs caused a further decrease in the glass transition temperature (94 °C for E″ and around 110 °C for tan δ). Moreover, the highest E″ values were noted for the PrIIPs membrane with the highest addition of 5 wt.% MWCNT-PrIIPs (almost double growth), which meant that it had the best resistance to elastic deformation. The increase in E′ and E″ with temperature suggested enhanced thermal-mechanical stability in hybrid materials. So, the introduction of MWCNT-REEIIP additives significantly reinforced the polymer matrix and improved strength, stiffness, and ductility—critical for membrane durability under mechanical and thermal stress.

**Figure 5:**
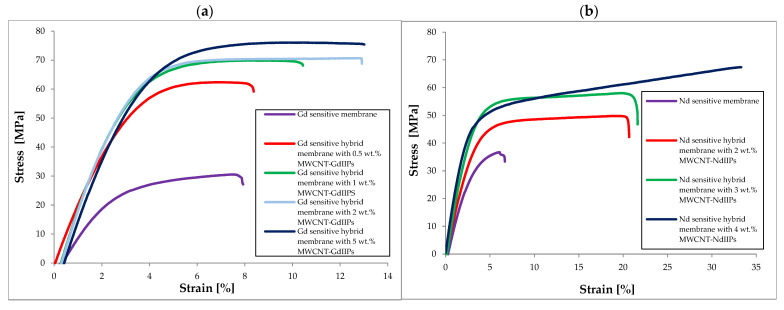
Dependency of elongation on standard force for: (**a**) Gd sensitive hybrid membranes with various additions of modified MWCNT-GdIIPs (from 0 to 5 wt.%), and (**b**) Nd sensitive hybrid membranes with various additions of modified MWCNT-NdIIPs (from 0 to 4 wt.%).

Due to the potential future applications of the newly developed adsorption membranes for the recovery of REEs from extracts, it was important to examine their mechanical properties, such as tensile strength (R_m_), Young’s modulus (E), and elongation at break. Selected results are presented in Table 1 and Figure 5. It was found that the mechanical properties of membranes consisting of two REEIIPs were comparable, with the Nd-selective membrane exhibiting slightly higher values of the tested parameters (from 10 to 20%). In addition, for both types of membranes, an increase in the values of the mechanical parameters was noted with an increase in the modified MWCNT addition, which might indicate good dispersion of the inorganic particles in the polymer matrix and appropriate interaction of the modified nanoparticles with the matrix based on the same IIPs. In the case of Nd-selective membranes, an increase in R_m_ by about 80%, E modulus by about 150%, and an almost 4-fold increase in elongation at break was noted, which indicated improved flexibility and toughness. In contrast, for Gd-selective membranes, the increase in R_m_ was almost 2.5-fold, 2-fold for Young’s modulus, and 60% increase for elongation at break. Higher values of Young’s modulus in the case of GdIIP hybrid membranes may be related to a higher final addition of modified MWCNTs (5 wt.%). An interesting observation was the gradual increase in E and R_m_ modulus in the case of NdIIP hybrid membranes, with the MWCNT addition and the rapid increase in the case of the GdIIP hybrid membranes with both types of membranes reaching similar values of the two mechanical parameters. The elongation at break value was almost 2 or even 3-fold lower for membranes based on GdIIPs. The observed differences may result from significant differences in the functionalization of MWCNTs with ionically imprinted polymers, wherein in the case of Gd the degree of functionalization was only 12%, while for Nd it was 29%. This may be related to the lower compatibility of MWCNT-GdIIP nanotubes with the polymer matrix, which may directly affect the lower values of elongation at break. It should also be noted that the use of an external magnetic field during membrane production had a positive effect on the dispersion of MWCNTs in polymer matrices, as well as on their mechanical properties. The improvement in mechanical properties may result from the increase in the density of the membrane material and the reduction in the mobility of polymer chains with the increase in the filler addition.

**Figure 6:**
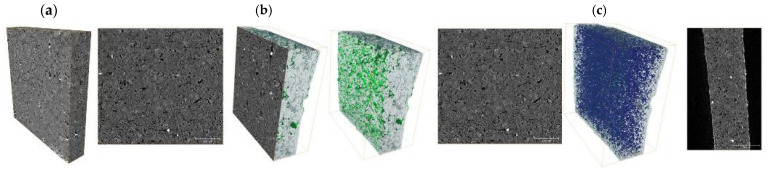
Results of X-ray tomography for a Nd sensitive hybrid membrane with 1 wt.% MWCNT-NdIIPs: (**a**) cross-section, (**b**) 3D model of bright phase, and (**c**) 3D model of pores and voids.

Some of the newly developed hybrid membranes were also analyzed using X-ray micro-tomography. The presence of several phases can be seen in the X-ray tomography images (Figure 6).

Namely, the dark gray matrix represents modified chitosan, in which there are lighter bands representing a higher density material, such as the added second NdIIP, and white inclusions represent modified nanotubes (Figure 6a). The following figures show cross-sections and 3D models of the light phase (green), which represents the distribution of MWCNTs in the polymer matrix (Figure 6b).

The next figures show cross-sectional views parallel and perpendicular to the membrane plane, with pores and voids marked in blue (Figure 6c). The membrane with the addition of MWCNT-NdIIPs was characterized by high porosity, in which the structure consisted of many individual pores. In the membrane with a smaller addition of nanotubes, there were mostly single pores, but as the MWCNT concentration increased, the porous structure changed and consisted of single pores that merged into larger longitudinal ones.

It could be seen from Figure 6a that the modified chitosan NdIIP was dispersed uniformly. While, after the addition of modified MWCNT-NdIIPs, they were dispersed homogeneously and created a developed 3D structure inside the polymer matrix. So, the obtained materials were characterized by a homogeneous internal structure and well-dispersed pore networks appropriate for transport and adsorption processes.

**Figure 7:**
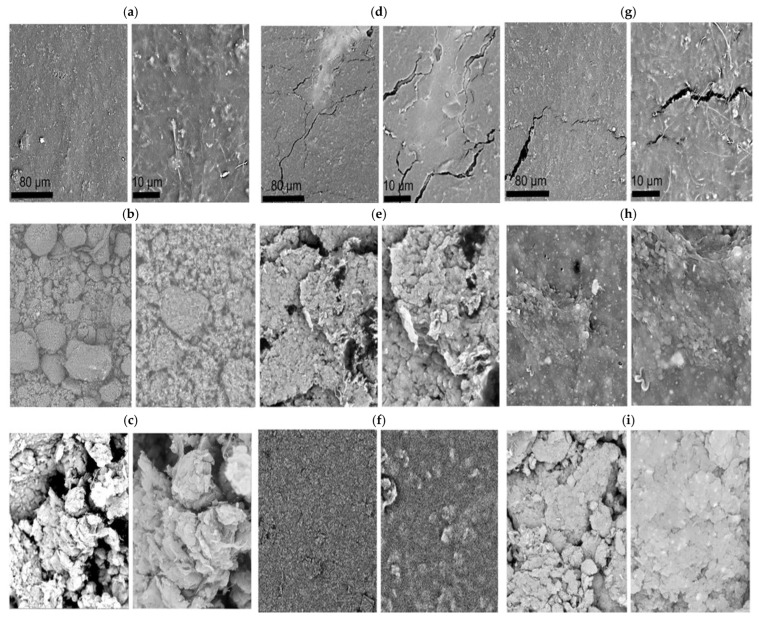
SEM images of REE sensitive adsorption membranes: (**a**) polymer membrane consisting of modified chitosan with 30 wt.% of PrIIPs, (**b**) hybrid membrane with the addition of 3 wt.% MWCNT-PrIIPs, (**c**) hybrid membrane with the addition of 4 wt.% MWCNT-PrIIPs, (**d**) polymer membrane consisting of modified chitosan with 30 wt.% of NdIIPs, (**e**) hybrid membrane with the addition of 2 wt.% MWCNT-NdIIPs, (**f**) hybrid membrane with the addition of 5 wt.% MWCNT-NdIIPs, (**g**) polymer membrane consisting of modified chitosan with 30 wt.% of GdIIPs, (**h**) hybrid membrane with the addition of 3 wt.% MWCNT-GdIIPs, and (**i**) hybrid membrane with the addition of 4 wt.% MWCNT-GdIIPs.

SEM analysis was also performed on the newly developed membranes, and the corresponding images are presented in Figure 7.

Figure 7 illustrates the suitable MWCNT dispersion that may be caused by the application of an external magnetic field during membrane production. Such a dispersion may result not only from direct interaction of the magnetic field with magnetic functionalized MWCNTs, but also from the interaction of the polymer matrix itself with MWCNTs modified with appropriate REEIIPs. The Nd- and Gd-sensitive adsorption membranes made of two polymers (Figure 7d,g) exhibited porous textures and small cracks, which could be caused by excessive drying of the membranes (during transport) before the SEM measurements. The membranes were usually stored in deionized water. SEM images of the hybrid membranes with the addition of MWCNT-REEIIPs showed a decent dispersion and the formation of an expanded 3D structure. The modification of MWCNTs with REEIIPs allowed obtaining membranes with a more uniform structure achieved by increasing the compatibility between the inorganic additives and the polymer matrix, even when the amount of the additive increased. In the case of membranes with nanotube additives without any modifications to the external structure, the compatibility between the polymer matrix of IIPs and the inorganic additive was found to be lower. Modification of the structure of nanotubes, especially by introducing REEIIP groups, resulted in a much better interaction between the inorganic and organic phases and more evenly distributed nanotube-polymer domains. The increase in MWCNT content led to denser, interconnected morphologies, potentially beneficial for barrier properties or selective adsorption. Modification with IIPs also provided certain benefits in the form of stronger interactions between those phases, which, in turn, translated into better separation properties of the membranes and an improved stability in the working environment.

**Figure 8:**
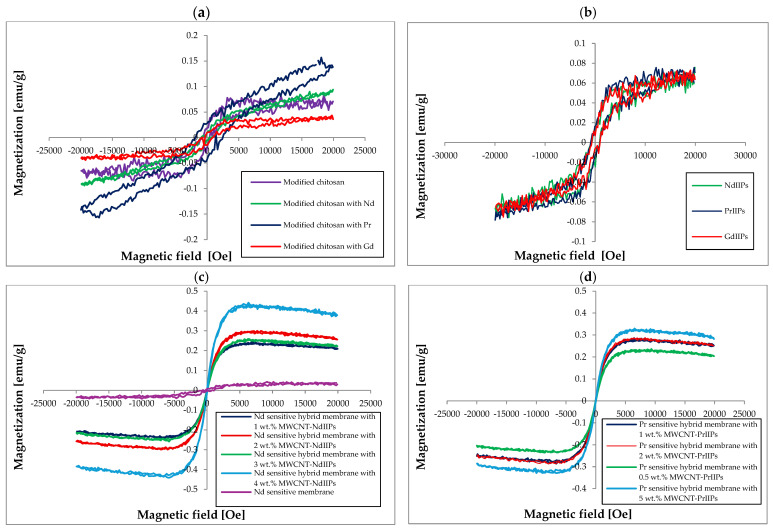

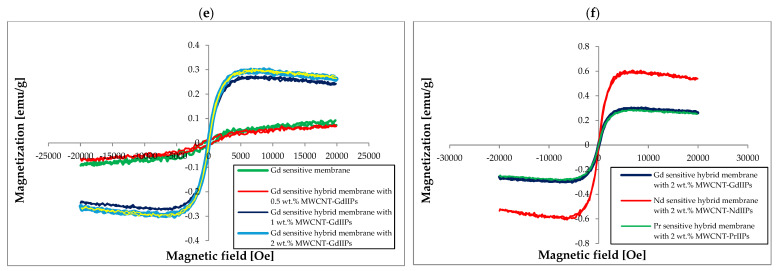
Magnetic hysteresis loops of polymers and REE-sensitive adsorptive membranes: (**a**) modified chitosan Schiff base, (**b**) copolymers of ternary complex and styrene-DVB, (**c**) Nd-sensitive adsorption membranes with various amounts of MWCNT-NdIIP additives, (**d**) Pr-sensitive adsorption membranes with various amounts of MWCNT-PrIIP additives, (**e**) Gd-sensitive adsorption membranes with various amounts of MWCNT-GdIIP additives, (**f**) comparison of various REEIIPs with a 2 wt.% addition of the appropriate MWCNT-REEIIPs.

The magnetic properties of newly developed adsorptive membranes were also tested, with the results presented in Table 2 and Figure 8. The hysteresis loops and magnetic parameters of REE ion-imprinted polymers indicated that they were ferromagnetic, but mostly with weak saturation magnetization (Ms ~0.04–0.15 emu/g). Their coercivity was found to be distributed in various ways (Hc ~300–850 Oe), thus indicating that the polymers were not magnetically soft materials. It was found that the introduction of REE ions changed the magnetic properties of the modified chitosan Schiff base, especially in the case of chitosan imprinted with Nd and Pr. Here, the saturation magnetization increased almost twice for Pr. At the same time, the changes in coercivity were found to be different, i.e., for the Pr-imprinted chitosan a decrease in coercivity was noted, while for the Nd- and Gd-imprinted chitosan coercivity increased. The remanence values for all imprinted chitosan samples were quite low. Similar results were obtained by Luan’s team, who studied the influence of metal doping on the magnetic properties of biphenyl networks. The introduction of metal ions induced various degrees of spin polarization, wherein, in the case of almost 100% polarization at the Fermi level, these materials may exhibit semi-metallic features, or in the case of incomplete spin polarization, exhibit antiferromagnetic properties [45]. In turn, in the works of Lebech and Rainford’s team, who studied the magnetic structures of Pr and Nd crystals, we can find mentions of the diverse influence of an external magnetic field depending on the direction of its interaction, where in some cases a significant increase in the values of induced magnetic moments or a decrease indicating high anisotropy of susceptibility was found [46].

The addition of modified MWCNTs significantly changed their magnetic properties. These membranes were produced from magnetic materials, both polymeric and MWCNTs, which interacted with each other in an external magnetic field. This had a significant influence on their magnetic, mechanical, and separation properties. It also had a direct impact on the dispersion of the inorganic additive and the properties of the newly developed membranes. It should be noted that for Gd-sensitive hybrid membranes a slight decrease in the value of magnetization saturation (Ms) was observed after the introduction of 0.5 wt.% MWCNT-GdIIPs (Figure 8e). For the remaining hybrid membranes, an increase in the value of this parameter was noted with the rising addition of modified MWCNTs: 3-fold for the Gd-sensitive, 4-fold for Pr-sensitive, and even 10-fold for the Nd-sensitive hybrid membranes with the highest filler content (Figure 8c,d). Comparing the hysteresis loops of hybrid membranes with 2 wt.% addition of modified nanotubes (Figure 8f) showed that their shape was similar. The magnetization values of the Pr- and Gd-selective hybrid membranes were very similar (over 0.3 emu/g), while that of the Nd-selective membrane was much higher (over 0.44 emu/g). This was reflected in the magnetic properties of the hybrid membranes based on these polymer matrices, indicating enhanced magnetic response via MWCNT incorporation. The value of the remanence (Table 2) remaining low across all samples (consistent with weak ferromagnetic behavior) slightly changed with an increase in the filler addition. On the other hand, the polymer membranes showed similar values of coercivity (765–850 Oe). Only the introduction of modified MWCNTs into the polymer matrix resulted in a decrease in the value of coercivity, which might be associated with creation in the outer magnetic field of more developed 3D structures in a polymer matrix. This phenomenon was confirmed by means of SEM analysis and, in particular, by X-ray micro-tomography. The decrease in coercivity might also be due to a reduction in insulation between nanoparticles due to mutual interactions during the production of hybrid membranes [47]. At the same time, the recorded values of coercivity for any of the membranes were not smaller than 22.86 Oe, which was indicative of weakly ferromagnetic properties. The largest decrease in the value of coercivity was recorded for the Gd-selective hybrid membranes, likely due to dispersion effects reducing anisotropy and domain wall pinning. However, in the case of Nd- and Pr-sensitive membranes, these decreases were much smaller, which might indicate a better insulation of nanoparticles, resulting from the greater MWCNT functionalization by NdIIPs and PrIIPs and their greater interaction with the polymer matrix. So, it could be seen that the hybrid structures allow the tuning of magnetic softness (lower coercivity) while enhancing overall magnetic responsiveness, desirable for magnetically driven separations or targeting [48,49].

The obtained hybrid adsorptive membranes were tested for their separation properties using a Sterlitech HP4750 (Sterlitech Corporation, Auburn, WA, USA) high-pressure stirred cell kit (pressure of nitrogen: 20–60 bar, temperature 20 °C, pH 7.5 of feed, which is synthetic coal fly ash extract, time 4–6 h) [50]. The recovery of adsorbed REE ions was performed in the same setup by the application of 50 mL of 1 M HCl after the separation process. It was found that the retention ratio increased with MWCNT-REEIIP addition, ultimately reaching 94.35%, 92.12%, and 90.11% for Nd, Pr, and Gd, respectively. While the recovery was around 89% and after 5 adsorption–desorption cycles was still 87%. The obtained membranes show a high rejection of Na, Mg, Ca, Al, Fe, and Si ions, which are components of the matrix in the extracts of coal fly ashes. The results obtained are comparable with usually used membrane techniques (recovery of REE ions over 89%). But it should be noted that the usually used membranes were applied for REE recovery from synthetic solutions, consisting of only a few REE ions and without matrix elements [8,16,51,52,53]. In the case of the usage of a feed consisting of REEs and matrix elements, such as Fe, Si, and Al, it was necessary to apply additional steps such as precipitation of the matrix ions, separation of monovalent ions, a microfiltration step, and finally separation of the rare earth elements by means of nanofiltration [54].

Our proposed solution enables the recovery of REEs from multicomponent, complex solutions corresponding to the composition of real coal fly ash extracts in the same reaction system. Furthermore, the use of highly selective and specific hybrid membranes enabled the separation of REEs with a high retention coefficient. Therefore, it should be stated that the tested hybrid membranes have the potential for further use in the recovery of rare earth elements from real coal fly ash extracts, and after introducing appropriate modifications, they will enable the production of single-component REE solutions in the future.

## 3. Materials and Methods

### 3.1. Reagents

Reagents of an analytical grade were used in this study (i.e., chitosan, sodium hydroxide, acetic acid, absolute ethanol, *o*-nitrobenzaldehyde (ON), glyoxylic acid, hydrazine monohydrate, zinc powder, Nd(NO_3_)_3_^.^6H_2_O, Pr(NO_3_)_3_^.^6H_2_O, Gd(NO_3_)_3_^.^6H_2_O, glutaraldehyde, hydrochloric acid, 5,7-dichloroquinoline-8-ol (DCQ), 4-vinylpyridine (4-VP), styrene, divinylbenzene (DVB), dimethyl sulfoxide (DMSO), and azobisisobutyronitrile (AIBN)) obtained from Sigma Aldrich (Poznań, Poland). Deionized water with conductivity 1 µS/cm was used in all solution preparation.

### 3.2. Ion-Imprinted Polymer Synthesis

**Figure 9:**
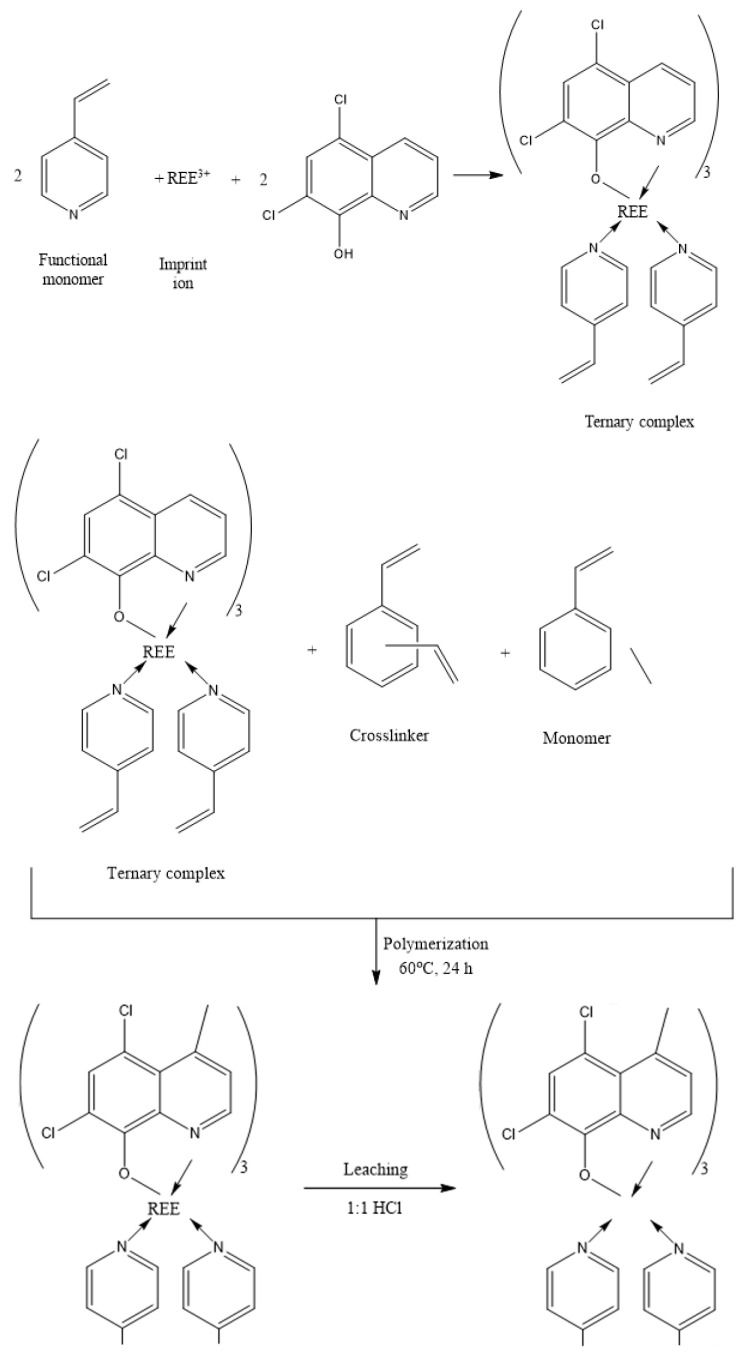
Scheme of the multi-stage reaction for obtaining the REEIIP through copolymerization of a complex between the REE(III), 5,7-dichloroquinoline-8-ol, and 4-VP and styrene-DVB as the monomer and crosslinker, respectively.

In this study, two types of polymers imprinted with selected rare earth metal ions were synthesized. The first multi-step procedure for obtaining modified chitosan was already described in the authors’ earlier work [50]. The second procedure was based on the copolymerization of a complex between REE(III), 5,7-dichloroquinoline-8-ol, and 4-VP and styrene-DVB as monomer and crosslinker (Figure 9). The reaction was performed in DMSO as a porogen. During this synthesis, the REE(III) salt (i.e., Nd(III) nitrate, Pr(III) nitrate, or Gd(III) nitrate) (0.2 mmol), was dissolved in 5 mL of DMSO and added dropwise to a solution (20 min) containing a mixture of DCQ (0.6 mmol) and 4-VP (0.4 mmol) in 5 mL of DMSO under magnetic stirring (600 rpm) in 25 °C. The ternary complex formation occurred in the magnetically stirred solution in 2 h, and after that styrene (20 mmol, 2.08 g, 2.3 mL), DVB (20 mmol, 2.60 g, 2.86 mL), and AIBN (0.6 mmol, 100 mg) were added. The mixture was stirred (600 rpm) for the next 2 h in 25 °C (until the AIBN was solubilized) and then cooled to 0 °C in an ice bath and then degassed with nitrogen for 15 min. The vial was next sealed and heated at 60 °C for 24 h under magnetic stirring (600 rpm). The obtained polymers were crushed and dried in an oven at 60 °C for 5 h.

### 3.3. Synthesis of MWCNTs

In our research, two types of iron-encapsulated MWCNTs (Fe@MWCNTs), namely commercial Nanocyl NC7000™ (Sambreville, Belgium) and synthesized in-house, were used. In-house Fe@MWCNTs were synthesized during 24 h via a catalytic chemical vapor deposition (c-CVD) protocol using the argon atmosphere (760 °C, argon 99.99% at the flow rate = 1.8 L/min, injection rate of the feedstock = 2.8 mL/h) as the growth environment, and a saturated solution of ferrocene in toluene as the feedstock. A toluene ferrocene solution (5.5 wt.%) dosed (2.8 mL/h) with a syringe pump, pre-evaporated in a heater (250 °C) controlled by an external unit, was injected into a stream of argon (1.8 L/min) as a carrier gas regulated by a mass flow controller. Hydrocarbon by-products were discharged through an oil scrubber. After synthesis, approximately 16 g of MWCNTs were obtained. To improve their REE affinity, capacity to adsorb REE ions, and further compatibility with polymer matrices—a few modifications were explored. Namely, at first, the oxidation of the carbon nanotube (CNT) surface was carried out to obtain CNT-OH or CNT-COOH, which were further functionalized with IIPs. The characteristic parameters of CNTs used in this study are given in Table 3. MWCNTs were oxidized using a mixture of concentrated sulfuric (VI) acid (95%) and concentrated nitric (V) acid (65%) during reflux. A total of 39 mL of concentrated sulfuric acid (95%) and 13 mL of concentrated nitric acid (65%) were added to 2 g of MWCNTs, which was then heated under reflux to boiling, maintained for 15 min, and then cooled down for 15 min. The prepared solution was poured into 500 mL of deionized water, and the obtained product was filtered under reduced pressure through a G4 sintered funnel and washed with 2.5 L of distilled water to obtain pH 7. The oxidized MWCNTs were dried in an oven to constant weight at 85 °C for 24 h, obtaining approximately 1.2 g of product.

MWCNTs were further modified in the reaction with 5,7-dichloro-8-quinolinol and appropriate REE nitrate in DMSO under reflux. A total of 50 mL of DMSO was added to 0.53 g of MWCNTs, and stirred on a magnetic stirrer (500 rpm) for 1 h. Then, 5,7-dichloro-8-quinolinol (0.645 g) and praseodymium nitrate (0.356 g) or neodymium nitrate (0.454 g) or gadolinium nitrate (0.430 g) were added and stirred at room temperature for 12 h. Then, styrene (2.3 mL) and divinylbenzene (2.9 mL) were added and stirred under nitrogen for 15 min. After that, azobisisobutyronitrile (0.110 g) was added and stirred for another 10 min at room temperature.

The mixture was heated at 60 °C for 24 h, then cooled to room temperature and filtered under reduced pressure. The modified MWCNTs were washed with DMSO (approximately 150 mL). Finally, the product was treated by 50 mL of 6 M hydrochloric acid solution for 12 h to remove the REE ions. The mixture was stirred in a magnetic stirrer (600 rpm) for 12 h. After this time, the final product in the form of CNTs modified with IIPs was filtered under reduced pressure and washed with deionized water until pH 7 and then dried at 50 °C to a constant weight.

### 3.4. REEIIP Membrane Preparation

Based on two synthesized polymers and modified CNTs, polymer membranes and hybrid membranes with filler addition ranging from 0.5 to 5 wt.% were prepared. In the next step, a 4 wt.% solution of the obtained modified chitosan was prepared in 2 wt.% acetic acid. Then, a 4 wt.% solution of neodymium, praseodymium, or gadolinium(III) nitrate was added to 20 mL of the chitosan solution and mixed on a magnetic stirrer for 12 h (600 rpm) and sonicated for another 2 h at 25 °C. This step was followed by the addition of 30 wt.% of the appropriate REEIIP, which was dispersed using a mechanical homogenizer for 0.5 h (15,000 rpm) in 25 °C. After that, functionalized MWCNTs (in the range from 0.5 to 5 wt.%) were added to this solution and then stirred for 12 h and sonicated for another 2 h at 25 °C. Then, 0.1 mL of a 50 wt.% glutaraldehyde solution was added, and the resulting solution was stirred for 0.5 h and sonicated for 15 min at 25 °C, before being cast on a leveled PTFE Petri dish and dried at 27 °C in a vacuum dryer to a constant weight. The modified MWCNTs were appropriately aligned and dispersed in the polymer matrix using an external magnetic field (100 mT) supplied by two ferrite magnets. The cast membrane was washed with deionized water and leached with a 1 M HCl solution for 24 h to remove REE ions and obtain imprinted cavities. Before carrying out the measurements, the membranes were soaked in a 1 M sodium hydroxide solution and then stored in deionized water.

### 3.5. Membrane Characterization

The synthesized IIPs, modified nanotubes, and hybrid membranes were also characterized by thermogravimetric analysis (TGA), scanning electron microscopy (SEM), vibrating sample magnetometry (VSM), X-ray diffraction (XRD), X-ray micro-tomography, and Fourier transform infrared spectroscopy (FTIR). The hybrid membranes were also studied in terms of their mechanical and rheological properties. During the performed property analysis, three identical membranes were tested by performing 2 measurements each, and the results were given as an average with standard deviation. The Lake Shore 7010 vibration magnetometer (VSM) (Westerville, OH, USA) was used for the analysis of magnetic properties. TGA was carried out using a TGA 8000 Perkin Elmer (Bridgeport, CT, USA) analyzer under an argon atmosphere (20 mL min^−1^) and using a heating rate of 20 °C min^−1^. In the case of unmodified MWCNTs, TGA in air (20 mL/min) was additionally carried out. The SEM analysis was performed using Phenom Pro Desktop SEM (Thermo Fisher Scientific, Waltham, MA, USA), with a 10-kV accelerating voltage. A thin layer of material was applied to carbon tape, then the excess sample was removed with compressed air. The samples were sputtered with gold, applying a layer of 5 nm. A Rigaku MiniFlex II diffractometer (Tokyo, Japan) with filtered copper radiation CuKα (λ = 1.54178 Å) was used for XRD analysis. The diffraction pattern was obtained in the range 2θ = 20–90° with a continuous recording at a speed of 0.4°/min. The mechanical measurements were carried out using the static testing machine Zwick/Roell Z050 (Ulm, Germany) (range 50 kN force). The dynamic mechanical analysis was conducted using TA Instruments Q800 DMA (New Castle, DE, USA). X-ray micro-tomography analysis was performed using X-ray micro-tomograph SkyScan 1174 (Bruker, Karlsruhe, Germany). FTIR spectra were acquired using a Specord M80 (Carl Zeiss, Jena, Germany) (KBr pellet—0.5 mg CNTs/1 g KBr, a 500–4000 cm^−1^ range).

## 4. Conclusions

The main objective of this study was the creation and characterization of a novel class of multifunctional hybrid membranes combining modified chitosan, functionalized multi-walled carbon nanotubes (MWCNTs), and rare earth element ion-imprinted polymers (REEIIPs), designed for selective adsorption and separation of lanthanide ions (Nd^3+^, Pr^3+^, Gd^3+^) from coal fly ash extracts. To evaluate potential future applications of proposed hybrid adsorptive membranes in industry, characterization of their magnetic, chemical, structural, mechanical, rheological, and thermal properties was conducted. The obtained membranes exhibit tunable magnetic properties through precise adjustment of MWCNT content, their functionalization, and the magnetic casting method (even a 10-fold increase in magnetic saturation and coercivity decrease with the rise in MWCNT addition). The proposed polymer matrix modifications and incorporation of REEIIP-functionalized MWCNTs simultaneously enhanced mechanical properties, like tensile strength and Young’s modulus (2-fold increase), and thermooxidative stability (Gd-sensitive membranes stable even up 730 °C). Performed structural (SEM and XRD) analysis confirmed homogeneous dispersion, phase compatibility, network integration, and formation of a complex 3D microstructure. While TGA and DMA analysis (almost a 2-fold increase in storage E′ and loss E″ modules) indicated improved operational stability of created hybrid membranes, which is significant for their future applications. Synthesized hybrid membranes showed a high rejection of matrix components and an increase in retention ratio with rising MWCNT-REEIIP addition, ultimately reaching 94.35%, 92.12%, and 90.11% for Nd, Pr, and Gd, respectively. This paper presents biopolymer-based, magnetically responsive, and REE selective and specific hybrid membranes that address the current need for sustainable, reusable, and high-performance materials applied in critical metal recovery and environmental protection. The studied properties of the newly synthesized adsorption membranes were extremely important in terms of their use for the recovery of Nd, Pr, and Gd from coal fly ash extracts with a rich composition, a complex matrix, and extreme pH ranges.

## Figures and Tables

**Table 1:** Mechanical properties of the newly developed membranes.

Membrane	R_m_ [MPa]	Young’s Modulus E [MPa]	Elongation at Break [%]
Nd sensitive membrane	36.72 ± 1.84	128.9 ± 6.3	6.68 ± 0.88
Nd sensitive hybrid membrane with 2 wt.% MWCNT-NdIIPs	49.80 ± 2.50	152.2 ± 7.4	20.68 ± 2.71
Nd sensitive hybrid membrane with 3 wt.% MWCNT-NdIIPs	58.03 ± 3.21	191.2 ± 9.3	21.63 ± 2.84
Nd sensitive hybrid membrane with 4 wt.% MWCNT-NdIIPs	67.37 ± 3.38	213.5 ± 10.4	33.30 ± 4.37
Gd sensitive membrane	30.55 ± 1.71	116.2 ± 7.4	7.93 ± 0.98
Gd sensitive hybrid membrane with 0.5 wt.% MWCNT-GdIIPs	62.37 ± 3.49	203.2 ± 9.9	8.37 ± 1.04
Gd sensitive hybrid membrane with 1 wt.% MWCNT-GdIIPs	69.88 ± 3.91	243.5 ± 15.6	10.44 ± 1.29
Gd sensitive hybrid membrane with 2 wt.% MWCNT-GdIIPs	70.68 ± 3.96	246.9 ± 15.8	12.91 ± 1.60
Gd sensitive hybrid membrane with 5 wt.% MWCNT-GdIIPs	76.06 ± 4.26	249.8 ± 16.0	13.01 ± 1.61

**Table 2:** Magnetic properties of synthesized polymers and Gd, Nd, and Pr sensitive membranes.

Membrane	Coercivity Hc [Oe]	Saturation Magnetization, Ms [emu/g]	Remanence [emu/g]
Modified chitosan	549.0 ± 27.9	0.085 ± 0.008	0.020 ± 0.001
Modified chitosan imprinted with Nd	736.4 ± 37.4	0.094 ± 0.009	0.010 ± 0.001
Modified chitosan imprinted with Pr	322.7 ± 16.4	0.157 ± 0.014	0.010 ± 0.001
Modified chitosan imprinted with Gd	804.5 ± 40.8	0.044 ± 0.004	0.010 ± 0.001
NdIIPs	781.8 ± 39.7	0.076 ± 0.007	0.020 ± 0.002
PrIIPs	895.4 ± 45.5	0.078 ± 0.007	0.020 ± 0.002
GdIIPs	818.2 ± 41.5	0.075 ± 0.007	0.020 ± 0.002
Nd sensitive hybrid membrane with 1 wt.% MWCNT-NdIIPs	133.09 ± 6.76	0.242 ± 0.022	0.003 ± 0.001
Nd sensitive hybrid membrane with 2 wt.% MWCNT-NdIIPs	126.62 ± 6.43	0.299 ± 0.028	0.003 ± 0.001
Nd sensitive hybrid membrane with 3 wt.% MWCNT-NdIIPs	65.75 ± 3.34	0.259 ± 0.024	0.002 ± 0.001
Nd sensitive hybrid membrane with 4 wt.% MWCNT-NdIIPs	48.87 ± 2.48	0.442 ± 0.041	0.009 ± 0.001
Pr sensitive polymer membrane	765.23 ± 38.85	0.079 ± 0.007	0.006 ± 0.001
Pr sensitive hybrid membrane with 0.5 wt.% MWCNT-PrIIPs	125.11 ± 6.35	0.236 ± 0.022	0.004 ± 0.001
Pr sensitive hybrid membrane with 1 wt.% MWCNT-PrIIPs	60.46 ± 3.07	0.286 ± 0.026	0.003 ± 0.001
Pr sensitive hybrid membrane with 2 wt.% MWCNT-PrIIPs	58.09 ± 2.95	0.288 ± 0.027	0.003 ± 0.001
Pr sensitive hybrid membrane with 5 wt.% MWCNT-PrIIPs	52.58 ± 2.67	0.329 ± 0.030	0.002 ± 0.001
Gd sensitive polymer membrane	811.61 ± 41.20	0.092 ± 0.008	0.008 ± 0.001
Gd sensitive hybrid membrane with 0.5 wt.% MWCNT-GdIIPs	808.92 ± 41.06	0.073 ± 0.007	0.010 ± 0.001
Gd sensitive hybrid membrane with 1 wt.% MWCNT-GdIIPs	155.10 ± 7.87	0.274 ± 0.025	0.007 ± 0.001
Gd sensitive hybrid membrane with 2 wt.% MWCNT-GdIIPs	40.27 ± 2.04	0.301 ± 0.028	0.005 ± 0.001
Gd sensitive hybrid membrane with 5 wt.% MWCNT- GdIIPs	22.86 ± 1.16	0.303 ± 0.028	0.005 ± 0.001

**Table 3:** Characteristics of the Fe@MWCNTs.

Property	Nanocyl NC7000^TM^ MWCNTs	In-House MWCNTs
Diameter [nm]	9.5	60–70
Length [mm]	1.5	>800
Aspect ratio	150	>12,000
Purity [wt.%]	90	96
Catalyst residue [wt.%]	<1 (+9 wt.% carrier Al_2_O_3_)	4.0

## Data Availability

The data presented in this study are available on request from the corresponding author.
